# Enhancing Electrical Conductivity of Stretchable Liquid Metal–Silver Composites through Direct Ink Writing

**DOI:** 10.1021/acsami.4c02466

**Published:** 2024-04-30

**Authors:** Wuzhou Zu, Hugo E. Carranza, Michael D. Bartlett

**Affiliations:** † Mechanical Engineering, Soft Materials and Structures Lab, Virginia Tech, Blacksburg, Virginia 24061, United States; ‡ Macromolecules Innovation Institute, Virginia Tech, Blacksburg, Virginia 24061, United States

**Keywords:** liquid metal, silver, material extrusion, DIW, stretchable electronics, multifunctional materials

## Abstract

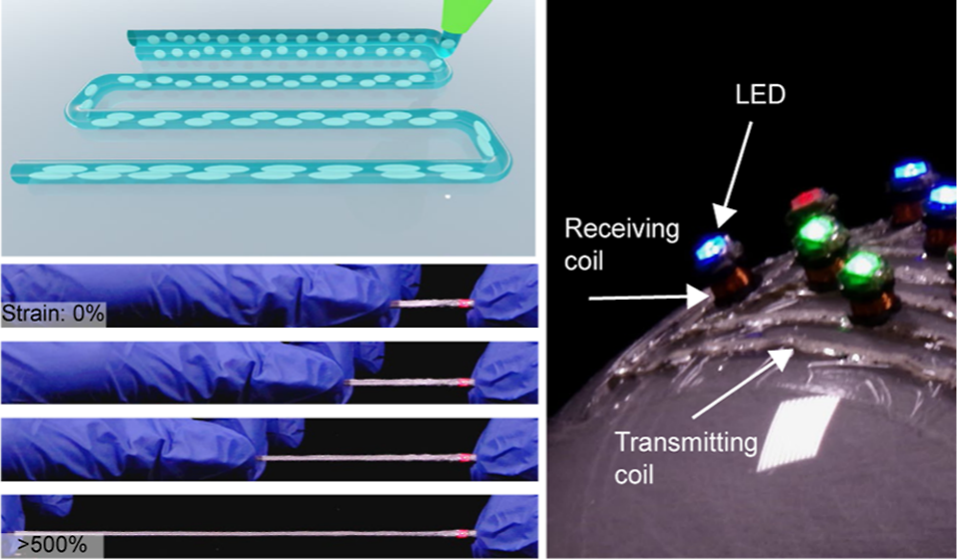

Structure–property–process relationships are a controlling factor in the performance of materials. This offers opportunities in emerging areas, such as stretchable conductors, to control process conditions during printing to enhance performance. Herein, by systematically tuning direct ink write (DIW) process parameters, the electrical conductivity of multiphase liquid metal (LM)-silver stretchable conductors is increased by a maximum of 400% to over 1.06 × 10^6^ S·m^–1^. This is achieved by modulating the DIW print velocity, which enables the *in situ* elongation, coalescence, and percolation of these multiphase inclusions during printing. These DIW printed filaments are conductive as fabricated and are soft (modulus as low as 1.1 MPa), stretchable (strain limit >800%), and show strain invariant conductivity up to 80% strain. These capabilities are demonstrated through a set of electromagnetic induction coils that can transfer power wirelessly through air and water, even under deformation. This work provides a methodology to program properties in stretchable conductors, where the combination of material composition and process parameters leads to greatly enhanced performance. This approach can find use in applications such as soft robots, soft electronics, and printed materials for deformable, yet highly functional devices.

## Introduction

There is a growing interest in soft and stretchable electronics because of their wide range of potential applications, such as wearable healthcare devices,^1−7^ soft sensors,^8−11^ electronic skins,^12,13^ and human–machine interfaces.^14−16^ Stretchable conductors are essential to the development of these electronics along with their future applications because of their unique ability to maintain high electrical conductivity while experiencing large mechanical deformations such as bending, twisting, and stretching. Gallium-based liquid metals (LMs) show properties of both fluids and metals,^17^ providing promising combinations of properties for stretchable conductors. Eutectic gallium–indium (EGaIn) has been a liquid metal of particular interest due to its low fluidic viscosity, negligible toxicity, and high electrical and thermal conductivity.^18,19^

One promising approach for soft and stretchable conductors is to incorporate LM droplets within soft elastomer matrices. The suspended mirco- or nanosized LM droplets inside the elastomer matrix can exhibit high thermal and electrical conductivity while maintaining the elastic properties of the host elastomer matrix. Recent studies have introduced LM soft composites with unique attributes, including self-healing,^20,21^ recyclability,^22^ programmable thermal conductivity,^23−25^ extreme stretchability,^26,27^ and good stability in underwater environments^28^ by varying LM volume loadings, processing methods, and host matrices. Multiphase systems where other particles are added to LM composites also provide a way to enhance functionality.^29,30^ This has been shown with solid fillers such as carbon nanotubes (CNT),^31^ silver (Ag) microflakes,^32−34^ Ag nanoparticles (AgNP),^35^ and poly(3,4-ethylenedioxythiophene) polystyrenesulfonate (PEDOT:PSS).^36^ Multiphase systems also provide a way to create composites which are electrically conductive as fabricated.^32−34,37−39^ Typically, composites with LM droplets alone need a secondary process, such as mechanical activation, to enable electrically conductivity.^23,40^ This is attributed to the lack of a percolated network during the fabrication of LM composites without secondary phases. Therefore, multiphase systems provide a route for a single process step to create percolated networks during fabrication, providing exciting possibilities for the on-demand creation of conductive materials. For example, seminal work in this area reported that an EGaIn coating could improve the electrical conductivity and mechanical deformability of Ag-based conductive circuits.^41^ By combining multiphase metallic conductors with tough and deformable polymers,^42−44^ soft and stretchable electronics can be fabricated with high, intrinsic electrical conductivity.

Although the advances in LM composites are encouraging, several common fabrication methods, such as screen printing, spray printing, or casting, typically lack tunability during material deposition. Several of these challenges can be overcome through emerging techniques in additive manufacturing, which enable digital programmability during printing. Among different material extrusion methods, direct ink writing (DIW) which prints a wide range of inks, from biomaterials to ceramics, is of great interest for printing soft composites.^45−48^ In a typical DIW process, the ink is selectively dispensed through a nozzle on the print bed, controlled by a command script. Recently, it has been shown that by controlling the process conditions, initially spherical LM droplets can be systematically elongated during DIW printing of LM composites. This enables the programming of LM microstructures for control of droplet orientation and aspect ratio for on-demand control of properties like thermal conductivity.^23^ However, this previous work focused only on LM composites. How process conditions control the ultimate structure of multiphase systems and how this influences electrical properties, is unknown.

Here, we show up to a 400% enhancement in electrical conductivity by tuning the DIW print velocity for multiphase liquid metal (LM)-silver stretchable conductors, resulting in printed conductors that exceed 1.0 × 10^6^ S·m^–1^. This is achieved by increasing the nozzle velocity relative to the extrusion velocity, which induces in situ alignment and elongation of the multiphase inclusions within the directly deposited soft conductors, leading to enhanced electrical conductivity. As nozzle velocity increases, the electrical conductivity of the as printed filaments increases. Through microstructural analysis, this increase in electrical conductivity is attributed to the improved alignment of Ag flakes and elongation of LM droplets within the polymer matrix. These DIW printed elastic conductors possess excellent combinations of programmable high conductivity, low modulus, and stable electrical response to mechanical deformation. To demonstrate the potential applications of these DIW printed elastic conductors, an electromagnetic induction coil for wireless power transfer is designed that is able to function under bending, twisting, and stretching. This work shows the importance of understanding the structure–property-process relationships in the creation of soft, functional materials. The ability to create as printed soft conductors with high electrical conductivity and stretchability can find use in soft robots, soft electronics, and printed materials for functional devices.

## Results and Discussion

### DIW Printing of Multiphase Functional Inks

**Figure 1:**
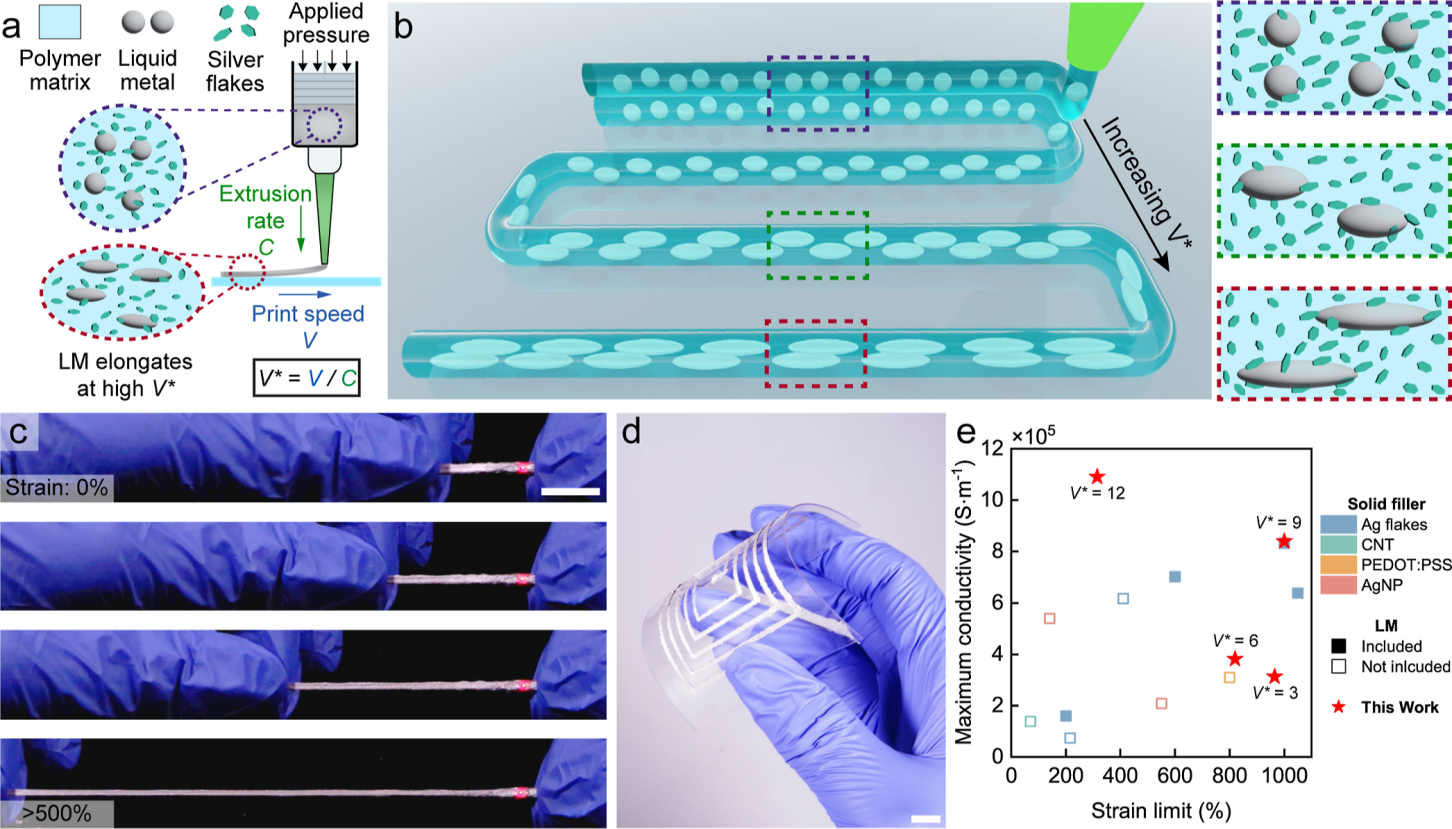
Direct in situ programming of the LM-Ag biphasic microstructure. (a) Schematic illustration of emulsion ink composition and printing process. (b) Schematic illustration of process control to tune the microstructure of LM droplets inside the composite. (c) Stretching a free-standing, as printed Ag-LM elastic conductor while an LED stays on. (d) Digitally printed spiral on a clear flexible PVC substrate. (e) Comparison between this work and other works on digitally printable elastic conductors.^31−35,49−53^ Scale bar: 10 mm.

The polystyrene-block-polyisoprene-block-polystyrene (SIS)-based emulsion ink contains Ag flakes as the solid conductive filler and EGaIn as the liquid conductive filler (Figure 1a). Material extrusion is performed by loading the ink into a desktop piston-driven DIW printer at a print height of 20 μm. While printing at a fixed height, the process parameters of interest are the extrusion rate *C* and the nozzle velocity *V*,^54,55^ which can be represented as a nondimensionalized nozzle velocity (V* = *V*/*C*). Under high V* (V* > 1), the ink deforms during extrusion. Such deformations break the oxide layer and elongate the LM droplets inside the ink. As V* increases, the higher deformation induced on the ink contributes to higher aspect ratios of the LM droplets. This printing-induced elongation is maintained by the rapid reforming GaO_*x*_ layer on the surface of LM droplets.^56^ By systematic control of V*, the printed trace width and layer thickness can also be controlled (Figure S1) and the aspect ratio of LM droplets is tuned in the emulsion ink and, therefore, program the properties of printed stretchable conductors (Figure 1b). These printed conductors are highly stretchable with high electrical conductivity. A single filament is robust enough to be removed from the print bed and stretched in a free-standing form to over 500% strain, while an integrated LED stays illuminated (Figure 1c). Further, it can be directly deposited on flexible substrates like poly(vinyl chloride) (PVC) and medical adhesives and stay bonded under mechanical deformation (Figure 1d). When compared to previous work on digitally printed stretchable conductors, the multiphase composites printed at high V* show excellent properties as shown in Figure 1e. A more detailed version can be found in Figure S2 and Table S1. Common solid fillers used to create stretchable conductors include Ag flakes, CNT, PEDOT:PSS, and AgNP. Ag flakes and CNT are the most widely used in the field of printed sensors because of their high aspect ratio.^57^ It is also noticed that by incorporating LM with solid fillers both the maximum strain and conductivity are improved. This plot shows the high strain limit and high conductivity of our Ag-LM composite and that by changing process conditions (V* = 12), the electrical conductivity of Ag-LM composite can increase up to 1.06 × 10^6^ S·m^–1^, one of the highest reported in printed multifunctional composites for stretchable electronics.

### DIW Process Tuning to Program Microstructure

**Figure 2:**
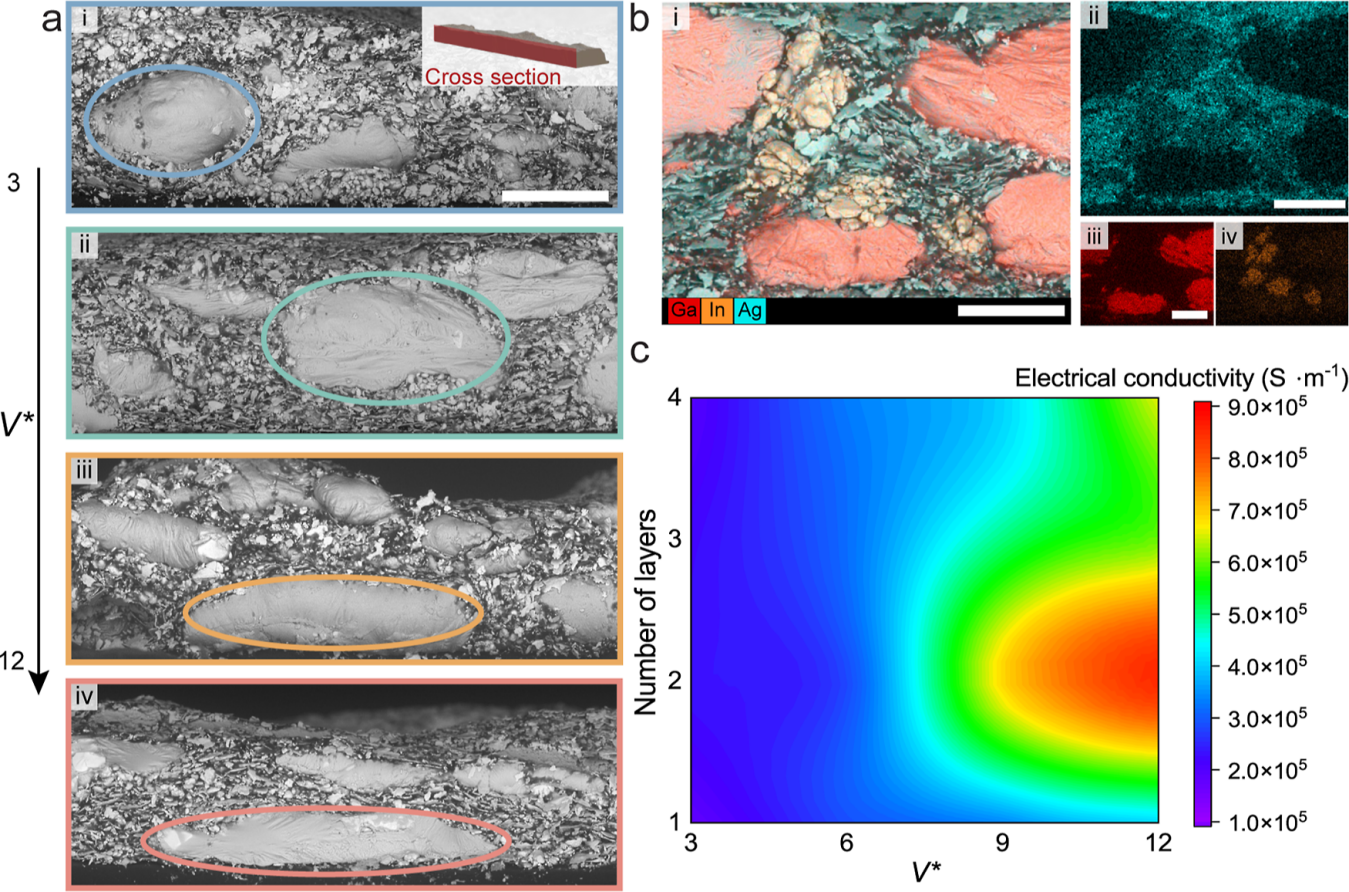
Process-structure–property relationships for DIW printed multiphase elastic conductors. (a) Microstructural analysis on the longitudinal cross-section of printed Ag-LM inks from V* = 3 to V* = 12 under SEM. Scale bar: 50 μm. (b) Elemental analysis through energy-dispersive spectroscopy (EDS) of the printed conductor. Scale bar: 25 μm. (c) Color map of the average electrical conductivity as a function of V* and number of printed layers.

To understand how the microstructure changes with different print conditions, a series of experiments were conducted by varying V* (3, 6, 9, and 12) and the number of layers printed (1–4) at a fixed print height of 20 μm. To understand how process conditions (V*) control the ultimate structure of a multiphase system and how this influences electrical properties, the longitudinal cross sections of two-layered traces printed with different V*s are examined under a scanning electron microscope (SEM). Figure 2a shows an increase of LM droplet aspect ratio as V* increases, similar to the findings in the previous work.^23^ Additionally, V* also affects the microstructure of the solid fillers. As V* increases, the Ag flakes become orientated in the print direction due to ink deformation during printing. A similar reorientation of Ag flakes in a Ag-only composite has also been reported in a viscoelastic, liquid-like polymer matrix under cyclic mechanical stretching.^58^ This resulted in effective and stable conductive pathways. In our multiphase composite, the solid and liquid phases deform and orient synergistically. Here, the elongation of LM droplets increases the volume of LM on the longitudinal plane, which appears to also align and stack the Ag flakes. This provides an efficient connection between the solid and liquid phases within the composite and provides a processing methodology to increase the electrical conductivity.

The elemental composition between solid and liquid conductive fillers is analyzed using EDS, as the map of the interested elements (Ga, In, and Ag) shown in Figure 2b. At the intersection between two neighboring LM droplets, obvious clusters of In are observed, regardless of the V*. These In clusters act as anchoring points between Ag flakes and Ga droplets because of the high affinity between Ag and In.^59^ Prior work summarized three phases of Ag–In: Ag_2_In (cubic), AgIn_2_ (tetragonal), and Ag_3_In (hexagonal).^60^ According to a more recent work,^38^ AgIn_2_ is formed when EGaIn and silver flakes are mixed at room temperature, which contributes to the uniform microstructure in this biphasic composite. These Ag–In intermetallic components are mainly present on the edges of the elongated LM droplets, as observed in the EDS analysis. Along the printed trace, these intermetallic anchors are widely distributed around elongated LM droplets, aiding in the continuous printing of the multiphase composite without notable phase separation.

To quantify improvements in the efficiency of electron conduction in the DIW printed elastic conductors, the electrical conductivity of each V* (3, 6, 9, 12) for different numbers of printed layers (1–4) is measured. As V* increases from 3 to 12, the largest increase in average conductivity occurs for the two-layered elastic conductor. Here, average conductivity increased from 2.34 × 10^5^ S·m^–1^ to 8.56 × 10^5^ S·m^–1^, with a maximum conductivity of 1.06 × 10^6^ S·m^–1^ for a single sample (Figures 2c and S3a). For all other numbers of printed layers, the electrical conductivity also increased.

Furthermore, by tuning the volume ratio of LM:Ag from 4:1 to 2:1 while maintaining the same total inclusion volume loading of 80%, electrical conductivity also generally increases as V* increases (Figure S3b). The high Ag content sets a high electrical conductivity for a low V*. Then by increasing V*, electrical conductivity generally increases, especially for 2 printed layers. The maximum conductivity for the higher Ag content (2:1) samples is 2.10 × 10^6^ S·m^–1^ at V* of 12. Compared to the 4:1 LM-Ag composite, the electrical conductivity for the 2:1 ratio increases less dramatically (≃ 50% increase). This is likely due to the decreased volume of liquid metal fillers that are able to deform and provide more efficient conductive pathways.

Based on the microstructures of the printed elastic conductors, the increase in conductivity is proportional to the increase in the elongation and alignment of LM droplets and Ag flakes (see Figure S4 for a plot of aspect ratio of elongated LM droplets versus electrical conductivity.). This improvement is attributed to a decrease in resistance for electrons to flow between the conductive inclusions. These process-structure–property relationships demonstrate how DIW processes can be used to control the multiphase composite microstructure, which can in turn enhance the electrical conductivity of printed elastic conductors.

### Mechanical and Electromechanical Properties of Printed Filaments

**Figure 3:**
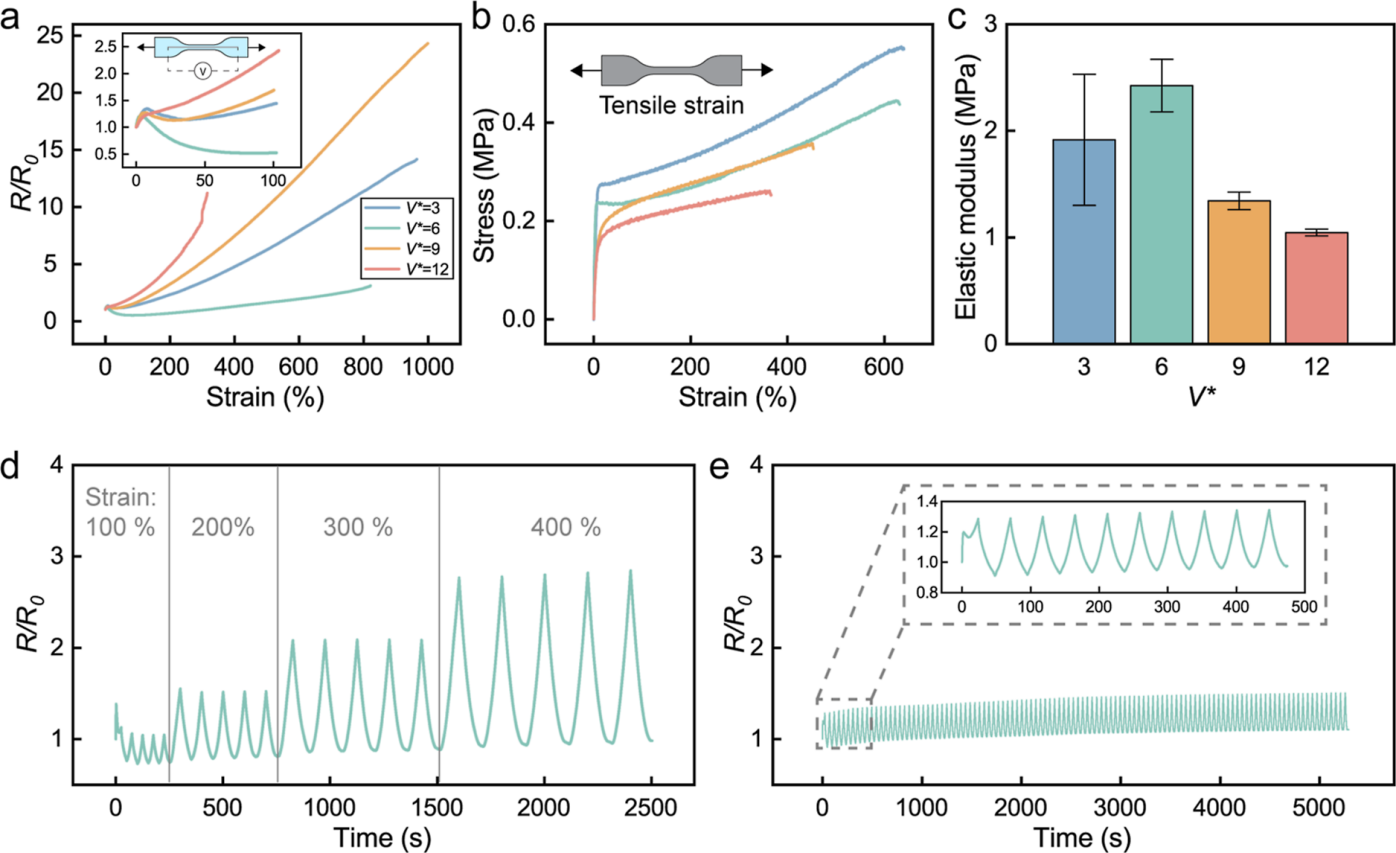
Mechanical and Electromechanical properties of DIW printed elastic conductors. (a) Electromechanical properties of V* = 3, 6, 9, 12. (b) Mechanical properties of conductors printed under different V*s. (c) Elastic modulus for different V* values. (d) Step cyclic test stretching an elastic conductor printed at V* = 6 from 100 to 400%, 5 cycles each. (e) Electromechanical coupling of the elastic conductor printed at V* = 6 under 100 cycles of stretching to 100% strain.

The process parameters are also found to influence the electromechanical behavior of the printed filaments. The electromechanical coupling is measured by monitoring the change in relative resistance (*R*/*R*_0_) as a function of uniaxial strain (Figure 3a), following similar procedures to prior work.^61,62^ The strain limit is set to 1000% during these tensile tests. The relative resistance of all V*s stays below 2.50 for 100% of strain. For the V* = 6, the relative resistance is as low as 0.52 at 100% of strain. Such low and stable relative resistance of V* = 6 continues as the strain goes beyond 100%. The relative resistance stays below 1.0 until 300% of strain and increases to 3.1 at its strain limit of 821% strain, which makes it a good candidate for elastic conductors. However, as V* increases to 9, the electromechanical coupling behavior changes where the relative resistance goes up to 24.2 at the 1000% strain limit. The V* = 12 is the outlier among these curves as the relative resistance goes up the fastest, and it shows the lowest strain limit.

The electromechanical coupling from 0 to 30% strain shows two distinct behaviors. First, the relative resistance increases steeply between 0 and 10% strain and then decreases at 30% strain for V* = 3, 6, 9. During the first 10% tensile strain, EGaIn droplets are enclosed inside a Gallium oxide. However, with increasing strain the brittle gallium oxide likely ruptures,^63^ and then these EGaIn droplets form additional conductive pathways with adjacent Ag flakes. These new conductive pathways result in increased electrical conductivity. After all of the new conductive pathways are formed, the relationship between the resistance and strain becomes more linear and predictable. However, this phenomenon is not noticed in V* = 12 specimens. A possible explanation is that the large surface area of elongated LM droplets in the V* = 12 specimen has already reached a critical percolation density of the conductive fillers, limiting the formation of new conductive pathways.

The mechanical behavior of free-standing as printed elastic conductors with V* = 3, 6, 9, 12 is shown in Figure 3b. After an initial elastic response, the stress versus strain curves show strain-hardening. The strain at break decreases as V* increases, going from around 600% for V* = 3 to 400% for V* = 12. The elastic moduli are calculated based on a linear fit of the first 10% strain, plotted in Figure 3c. The elastic modulus as a function of V* is lowest for V* = 12 at 1.04 MPa and highest for V* = 6 at 2.42 MPa. The compliance and stretchability of these printed elastic conductors are sufficient for a range of soft technologies, which will be demonstrated later in the paper for applications of wireless power transfer.

Stretchable conductors must maintain stable conductivity under a cyclic loading. The relative resistance is also tested under two cyclic loading profiles: 100% strain increment to 400% strain (Figure 3d) and 100 cycles of 100% strain loading (Figure 3e). During the first set of 100% strain loading cycles, the relative resistance decreases 20% and remains stable after new conductive pathways are formed. Among the sets of 100, 200, 300, and 400% strain cycles, there is negligible change in the relative resistance between the first and last cycle during a set. For example, under the 400% strain cycles, the relative resistance while fully stretched increases by only 0.07 from the first loading cycle of this set. Further, the change in resistance of the sample at 0% strain is also negligible as *R*/*R*_0_ = 0.98 after four sets of incremental tensile loading cycles. This stability in electrical properties under mechanical deformations is also presented in the consecutive loading test, as shown in Figure 3e. Similarly, there is no significant change in resistance during 100 cycles of 100% strain loading. There is a 10% increase in resistance at 0% strain and 17% increase at 100% strain when comparing between the initial and final loading cycle.

### Demonstration of the Printed Elastic Conductors

**Figure 4:**
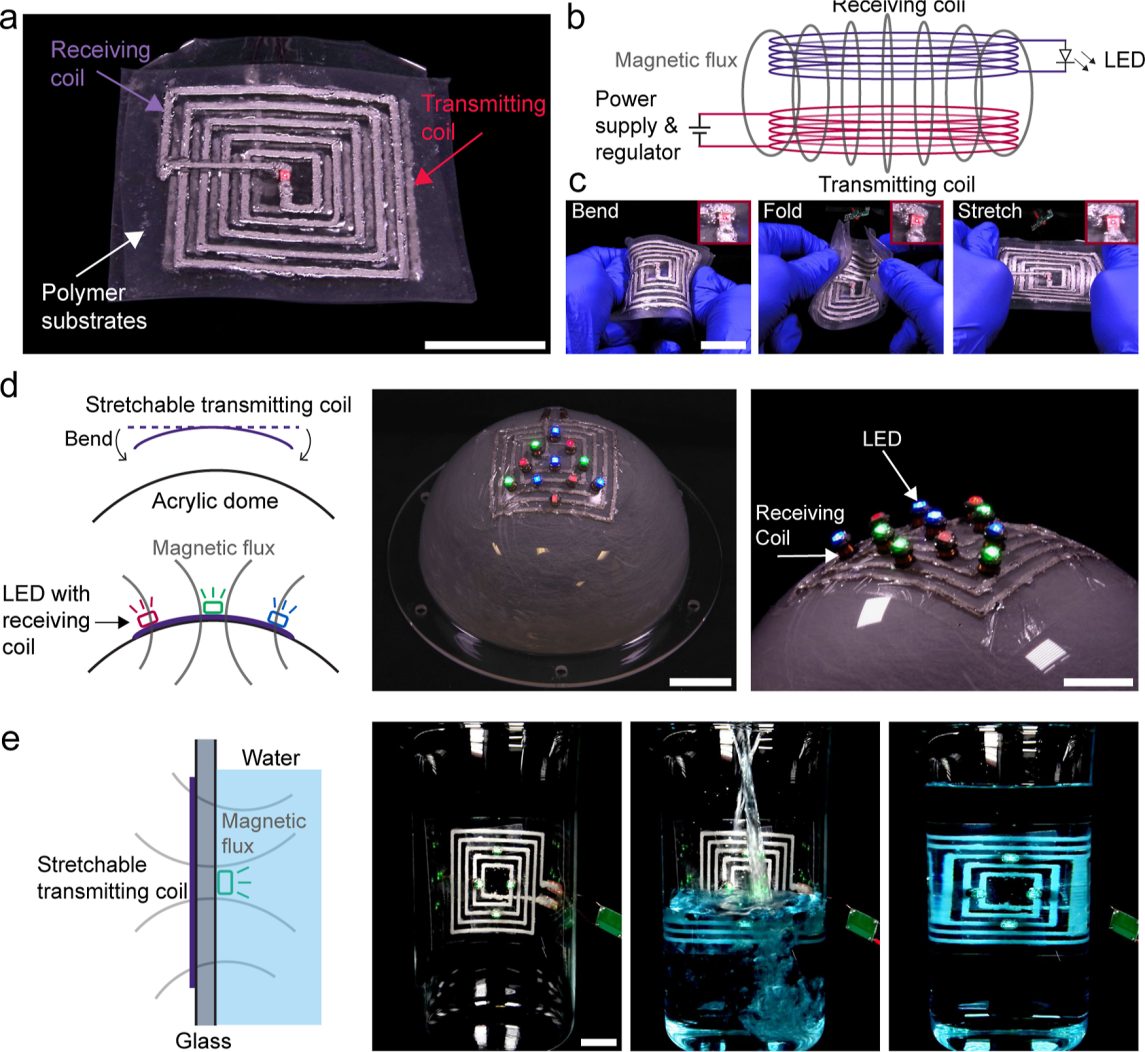
DIW printed flexible and stretchable inductive coupling coils. (a) Printed set of inductive coupling coils on SIS substrates. (b) The general principle of inductive coupling. (c) The coils can go under bending, folding, and stretching. (d) Stretchable coil adhered to the acrylic dome. LED with embedded receiving coil can be powered up at different angle on top of the dome. (e) Wireless power transfer through water. Scale bar: 10 mm.

To demonstrate the LM-Ag-SIS composite printed at high V* as a stretchable electrical conductor, a set of multilayered electrical circuits are designed and printed that are soft, flexible, and stretchable as inductive coupling electric coils (Figure 4a). These coils are able to transfer power wirelessly on irregular surfaces because of their combination of high conductivity induced by high V* during printing and high tolerance to mechanical deformation while maintaining a stable interconnection with the rigid LED component (Figure S5). These coils are printed following a rectangular concentric circular pathway with V* = 6 on stretchable SIS substrates. All of the printing conditions are the same as those specimens prepared for the characterization tests. A schematic illustration of the principle of inductive coupling is shown in Figure 4b. These coils are printed on a transparent PVC substrate with the V* of 6 because of its most stable electromechanical coupling under various mechanical deformations as indicated in Figure 3a. When the receiving coil is placed above the transmitting coil, the red LED in the center powers up wirelessly. Even when the set of coils undergoes various mechanical deformations such as bending, folding, and stretching, the LED still functions (Figure 4c). The close-up pictures presented as insets in Figure 4c demonstrate continuous power transfer between the coils under mechanical deformations.

To show the printability on various surfaces, flexible transparent sheets or films such as PVC sheets, medical adhesive (tegaderm), and PET films are also used as substrates. In Figure 4d, a transmitting coil printed on a medical-grade adhesive film is bonded to the irregular shaped surface of an acrylic dome with significant curvature. Wrapping spheres is difficult or impossible for rigid wireless power transfer coils made of solid metals like copper without additional design or fabrication steps. However, the low modulus and stretchability of the LM-based composite on the medical adhesive enable conformal contact across the curved surface. An array of LEDs with embedded receiving coils can be powered when placed at different angles and locations on the dome, demonstrating effective inductive power transfer across the surface of the curved dome. In addition, wireless underwater lighting is also demonstrated through stretchable inductive coupling coils. Figure 4e shows that by attaching a transmitting coil to the outside of a regular glass beaker, the LED embedded with the receiving coils inside the beaker can still light up under water. These demonstrations together demonstrate the ability to effectively transfer power through different media through the LM-Ag-SIS composite. This is attributed to its unique combination of high conductivity, stretchability, and stable electromechanical coupling induced by a systematically designed DIW printing strategy.

## Conclusions

This work demonstrates the effect of process on the properties of the DIW printed LM-Ag-SIS composite. By systematic tuning of the process conditions, multiphase LM composites form an engineered conductive network with programmable electrical properties. This is achieved by increasing the nondimensionalized nozzle speed V*, inducing in situ elongation, coalescence, and percolation of these multiphase inclusions that plays an important role in the final properties of this multifunctional composite. These DIW printed elastic conductors showed an enabling combination of high electrical conductivity, a strain limit, and stable electromechanical response. This was demonstrated through a wireless power transfer system that could readily confirm to challenging spherical surfaces with transferring power to LEDs. The work presented here takes advantage of the unique ability of liquid metal droplets to elongate under DIW printing. By utilizing this effect in a multiphase system with Ag flakes, the electrical conductivity of digitally printed elastic conductors is dramatically increased without other postprocessing methods such as sintering or chemical treatment. These results can inspire future work in developing effective strategies to couple materials and processing to enable the enhanced performance of soft and stretchable conductors for applications in soft electronics, soft robotics, and multifunctional structures.

## Experimental Section

### Ink Preparation

LM-Ag-SIS emulsion ink is fabricated by first dissolving the Poly(styrene-*b*-isoprene-*b*-styrene) (SIS) (14 wt % styrene; Sigma-Aldrich) pellets in toluene at a ratio of 2:3 by weight. Eutectic gallium indium alloy (Ga/In in the weight ratio of 3:1) droplets are added directly to the SIS emulsion. Ag flakes (SF94; Ames-Goldsmith) are then added as solid conductive fillers in addition to EGaIn to help construct conductive pathways inside the composite. The first step of mixing is to hand mix until a silvery and shiny suspension is obtained. Afterward, the ink is further mixed inside a centrifuging mixer (Speedmixer; Flacktek). Lastly, the ink is transferred into a 10 mL medical syringe and mounted on the DIW printer (Engine SR; Hyrel). After the solvent completely evaporated in a 40 °C oven for an hour, for the 4:1 ratio of LM:Ag, the printed trace consists of 20 vol % of SIS, 64 vol % of EGaIn, and 16 vol % of Ag flakes. For the 2:1 ratio of LM:Ag, the printed trace consists of 20 vol % of SIS, 53 vol % of EGaIn, and 27 vol % of Ag flakes.

### Ink Printing

The first step of printing is calibrating the DIW printer by leveling the print bed and adjusting the print height between the nozzle and the substrate on the print bed. Print nozzle inner diameter is 0.838 mm (18 Gauge). Different substrates were used during the characterization and demonstration, including polyethylene terephthalate (PET) films, PVC films, and SIS sheets. The GCode used for printing is generated by a written MATLAB script to have full control of the applied pressure and printing speed. As a result, the extrusion velocity *C* is fixed to be 4.1 mm·s^–1^ and the print head velocity *V* is set to 12.3, 24.6, 36.9, and 49.2 mm·s^–1^ for V* = 3, 6, 9, and 12, respectively, as summarized in Table S2. The print height is set to 20 μm for each layer during printing.

### Electron Microscopy and Elemental Analysis

The microstructure of the samples is characterized by a SEM (IT-500HR; JEOL) instrument equipped with an EDS detector (Ultim Max100; Oxford Instruments). To analyze the cross section, the sample is submerged in a glass dish filled with liquid nitrogen for 120 s and cut along the printing direction with a scalpel blade. The images were collected in backscattered electron (BSE) mode under low vacuum (50 Pa). The colormap is built from the EDS scanning on the cross-sectional surface to analyze the element distribution.

### Electrical Characterization

The electrical conductivity σ is calculated as , where *R* is the electrical resistance, *A* is the cross-sectional area, and *l* is the length of the specimen. Each sample in the conductivity measurement is printed with a length of 60 mm and a number of layers increasing from 1 to 4. The electrical resistance *R* is measured from a digital desktop source measuring unit (SMU) (2450; Keithley) using the four-point probe method. The cross-sectional area *A* is also directly measured from a 3D surface profiler (VK-X3000; Keyence) with a laser confocal scan approach, as shown in Figure S6.

### Mechanical and Electromechanical Characterization

In mechanical characterization, a rectangle with a 50 mm width and 60 mm length is printed with V* = 3, 6, 9, or 12 on a PTFE sheet. The standard dogbone shape (ASTM D412-C, 1/2 scale) is cut from the printed film using a manual die cut. In electromechanical characterization, a 45 mm long trace is printed on a cast SIS substrate, and the same dogbone shape is made using a CO_2_ laser cutter (VLS4.75; Universal Laser Systems). The sample is tested on an Instron 5944 universal testing machine with a 10 N load cell at an extension rate of 1 mm·s^–1^. Outside from the test grips, the electrical properties are acquired from the same SMU (2450; Keithley) used in conductivity tests that synchronizes via digital I/O channels. The results from these electromechanical characterization tests are summarized for the comparison visualized in Figure 1e. The elastic modulus is calculated from the slope of the stress–strain curve up to 10% that was obtained from mechanical characterization.

### Demonstration

A wireless charging development PCB is used to demonstrate the wireless power transfer capability of the printed coils. On the PCB, a wireless charging controller (XKT-001; Xingketai Electronics) is used to regulate the operating frequency. The PCB is connected to the printed coil by soldering two copper electrodes to the power supply and ground terminals. These two electrodes are then connected to the printed coil before the toluene evaporates. An extra layer of copper tape is also sealed to secure the electrical connection. To power the transmitting module, a 5 V DC voltage is supplied from a benchtop DC power supply. The LED with receiving coils was purchased from Adafruit and used as-is to receive the magnetic flux from custom printed transmitting coils. The substrates used for printing are PVC film and medical grade adhesive (Tegaderm; 3 M) purchased and used as received.

## Data Availability

The data that support the findings of this study are available from the corresponding author upon reasonable request.

## Supporting Information Available

Movie depicting stretching the printed elastic conductor (MP4)
Movie depicting powering LEDs wirelessly on a curved surface (MP4)
Movie depicting powering LEDs wirelessly on a curved surface underwater (MP4)
Print resolution of *V**; electrical conductivity analysis between two compositions; properties summary of recent works on digitally printable elastic conductors; mechanical durability of LED interfacing; list of print parameters of *V**; and cross-sectional area measurement (PDF)
