# Supplementary material for: Enhancing Electrical Conductivity of Stretchable Liquid Metal–Silver Composites through Direct Ink Writing
Source: ACS Appl Mater Interfaces. 2024 Apr 30;16(18):23895–903. doi: 10.1021/acsami.4c02466 (PMC11082841; doi:10.1021/acsami.4c02466)
Supplement: Supplementary file 4 — am4c02466_si_004.pdf [file am4c02466_si_004.pdf]

Supporting Information

Enhancing electrical conductivity of stretchable liquid metal-silver composites through direct ink writing

Wuzhou Zu[1], Hugo E. Carranza[1], and Michael D. Bartlett[1,2*]

[1]Mechanical Engineering, Soft Materials and Structures Lab, Virginia Tech, Blacksburg, VA 24061, USA.

[2]Macromolecules Innovation Institute, Virginia Tech, Blacksburg, VA 24061, USA.

[*]Corresponding author email: mbartlett@vt.edu

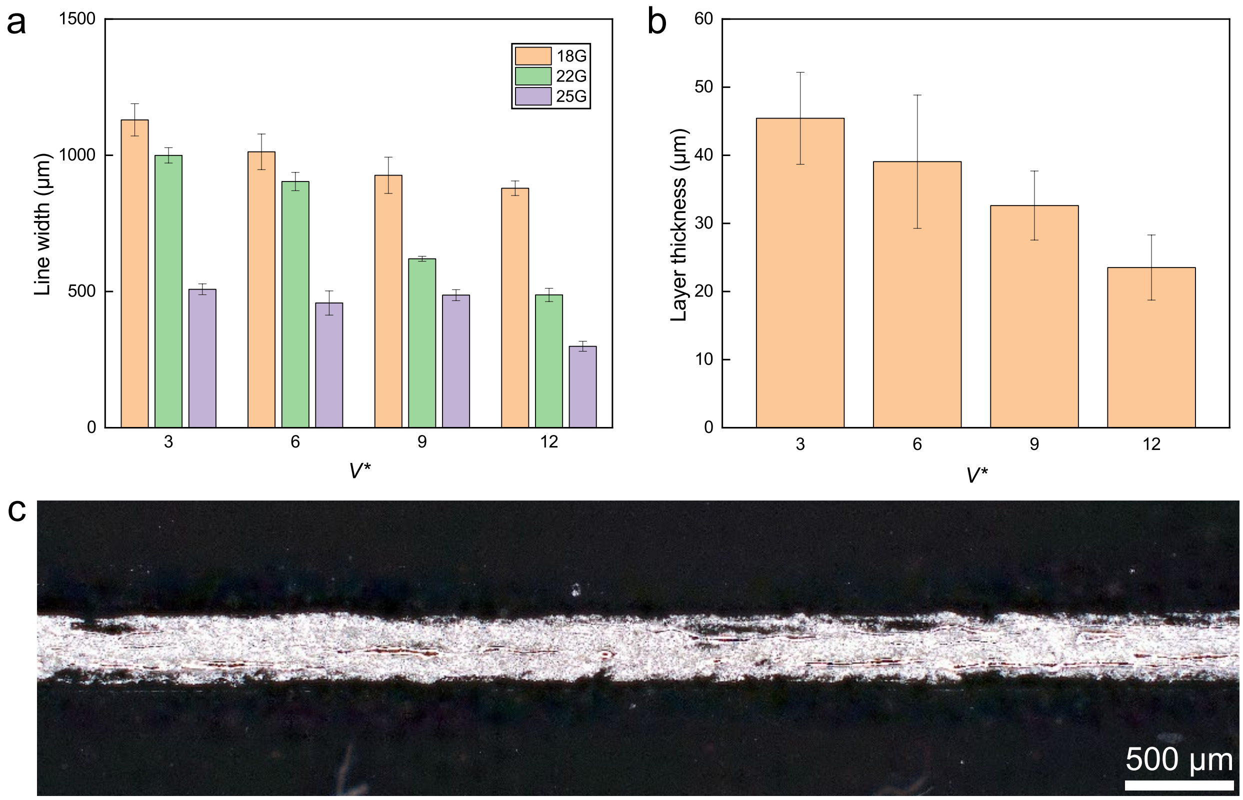

Figure S1 Printing resolution. a) Trace width as a function of V^* when printing with different sizes of nozzles: 18G (inner diameter of 838μm), 22G (413 μm), and 25G (260 μm). b) Layer thickness decreases as V^* increases. c) Micrograph of a trace printed with a 25G nozzle at $V^* = 12$.

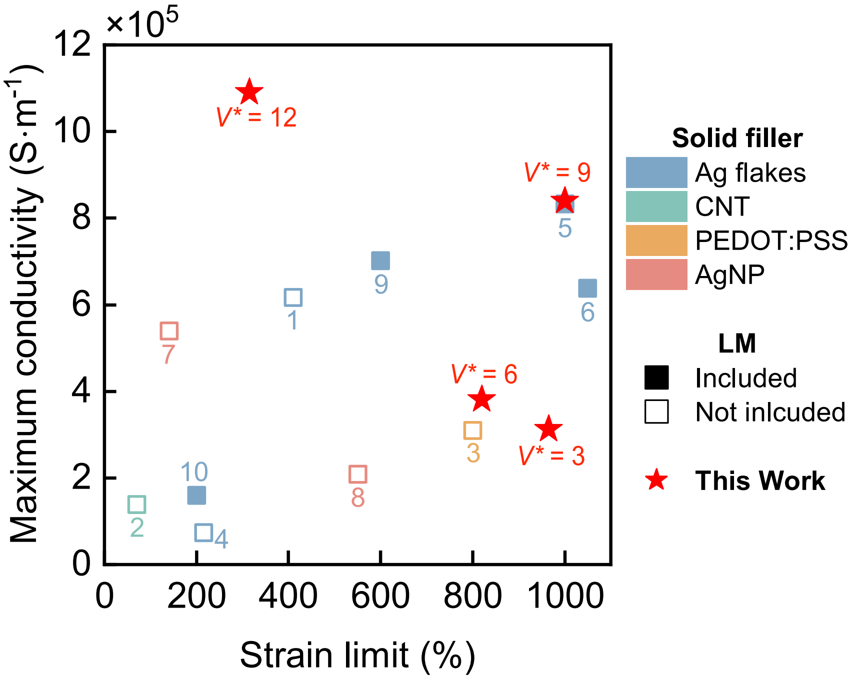

Figure S2 Recent works on digitally printable elastic conductors.

Key	**Strain limit** (%)	**Maximum conductivity** (S·m^{-1})	**LM**	**Solid filler**	**Reference**
1	410	6.17×10^5	Not included	Ag flakes	Matsuhisa 2017[49]
2	70	1.39×10^5	Not included	CNT	Lee 2019[31]
3	800	3.10×10^5	Not included	PEDOT:PSS	Wang 2017[50]
4	215	7.38×10^4	Not included	Ag flakes	Matsuhisa 2015[51]
5	1000	8.33×10^5	Included	Ag flakes	Wang 2018[33]
6	1050	6.38×10^5	Included	Ag flakes	Zu 2022[32]
7	140	5.40×10^5	Not included	AgNP	Park 2012[52]
8	550	2.08×10^5	Not included	AgNP	Song 2017[35]
9	600	7.02×10^5	Included	Ag flakes	Lopes 2021[34]
10	200	1.60×10^5	Included	Ag flakes	Carneiro 2023[53]
11	315	1.09×10^6	Included	Ag flakes	This work, $V^* = 12$
12	1000	8.39×10^5	Included	Ag flakes	This work, $V^* = 9$
13	820	3.81×10^5	Included	Ag flakes	This work, $V^* = 6$
14	965	3.12×10^5	Included	Ag flakes	This work, $V^* = 3$

Table S1 List of references in Figure S2.

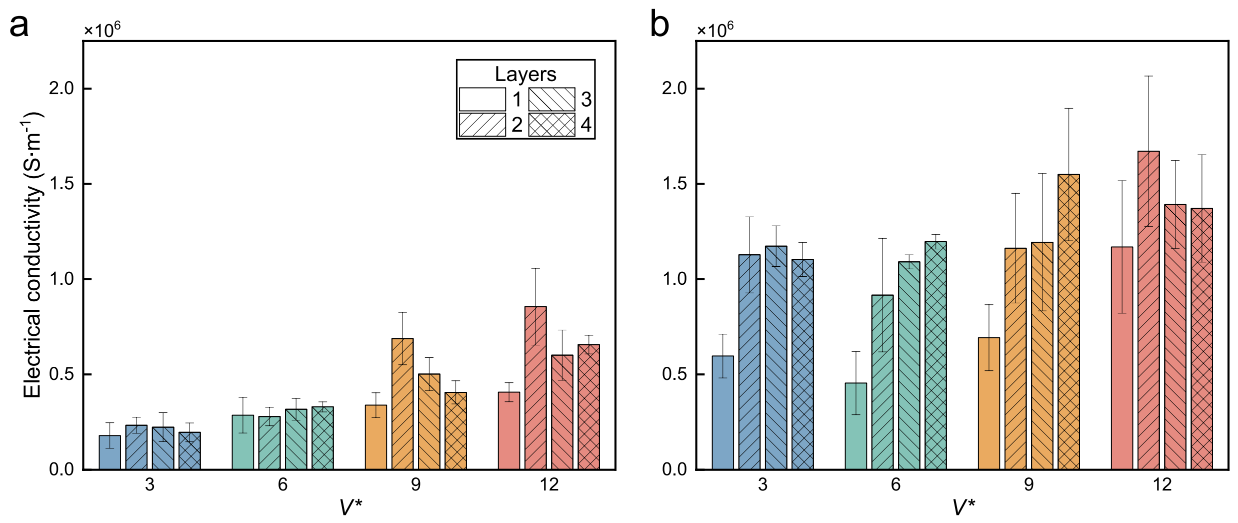

Figure S3 Electrical conductivity of printed filaments with different process conditions. a) Electrical conductivity for different printed layers as a function of V^*. The LM to Ag volume ratio is 4:1. b) Electrical conductivity for different printed layers as a function of V^*. The LM to Ag volume ratio is 2:1.

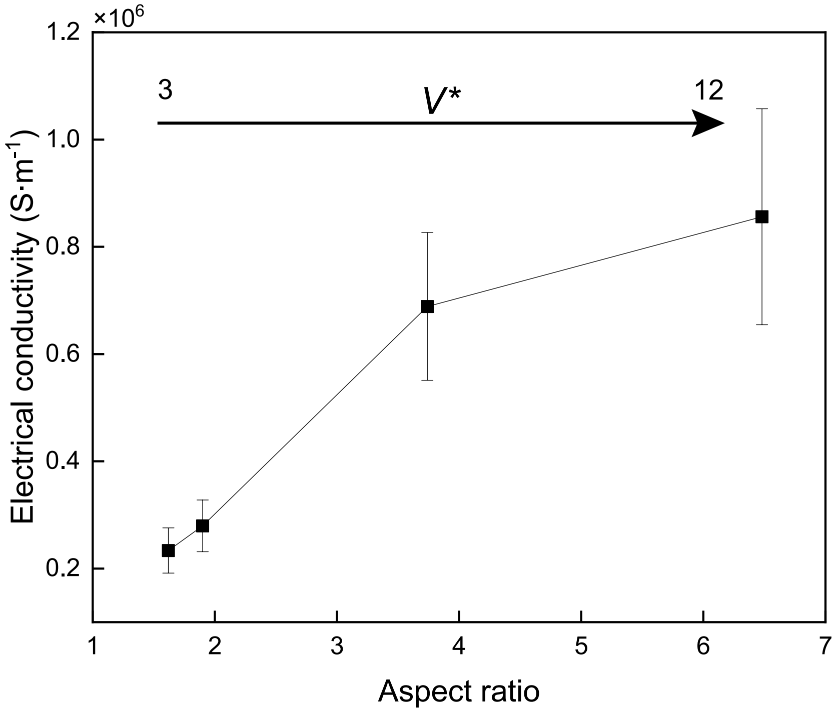

Figure S4 Electrical conductivity as a function of LM aspect ratio for two printed layers with the 4:1 LM:Ag composition. The data points represent V^* values of 3, 6, 9, and 12 going from low to high aspect ratio.

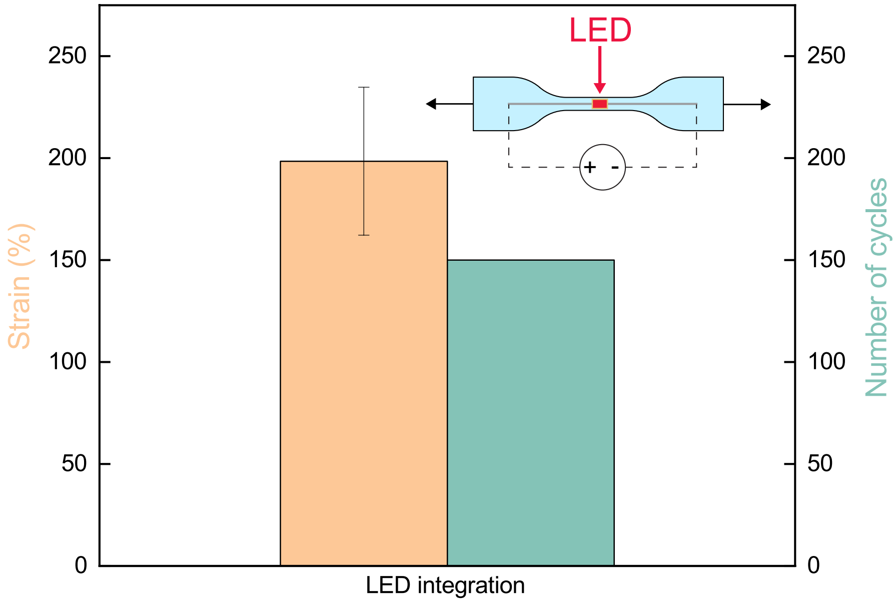

Figure S5 Mechanical durability of an LED integration on a printed traces with 4:1 LM:Ag composition, V^*=6, and two printed layers.

V^*	**Extrusion rate** C (mm·s^{-1})	**Nozzle velocity** V (mm·s^{-1})
3	4.1	12.3
6	4.1	24.6
9	4.1	36.9
12	4.1	49.2

Table S2 List of V^*s and their corresponding extrusion rate C and nozzle velocity V in mm·s^{-1}.

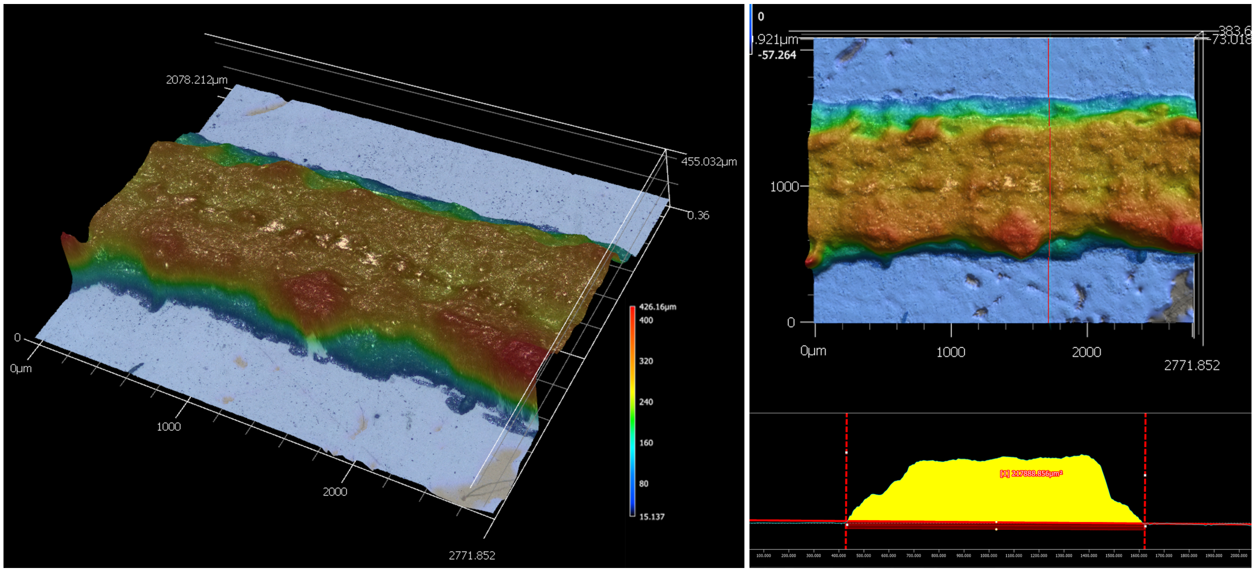

Figure S6 Representative cross-sectional analysis used for calculating electrical conductivity.

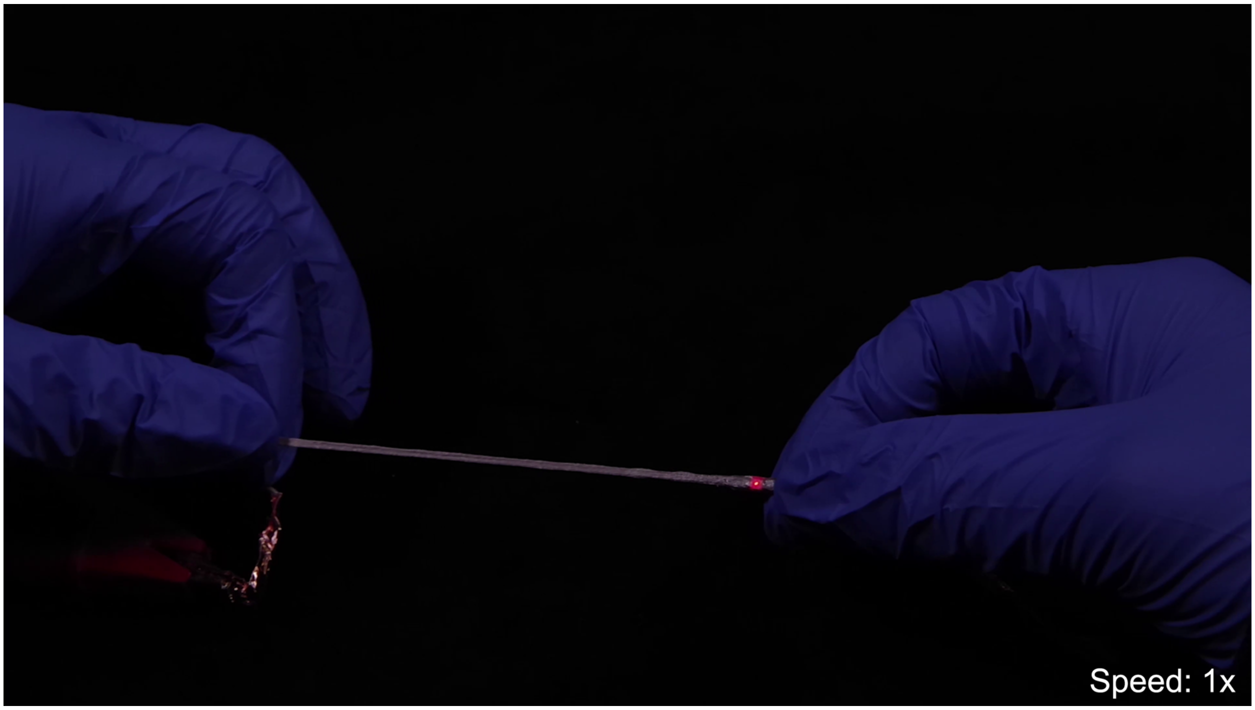

Movie S1 Stretching the printed elastic conductor.

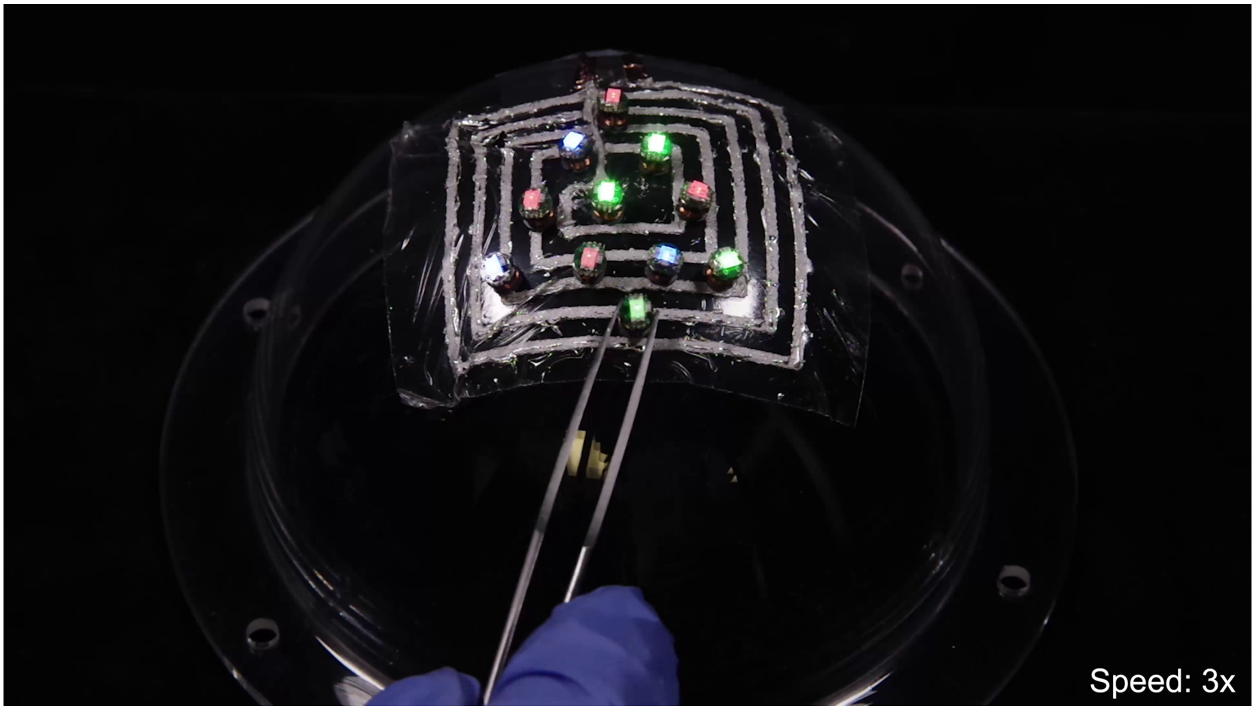

Movie S2 Powering LEDs wirelessly on a curved surface.

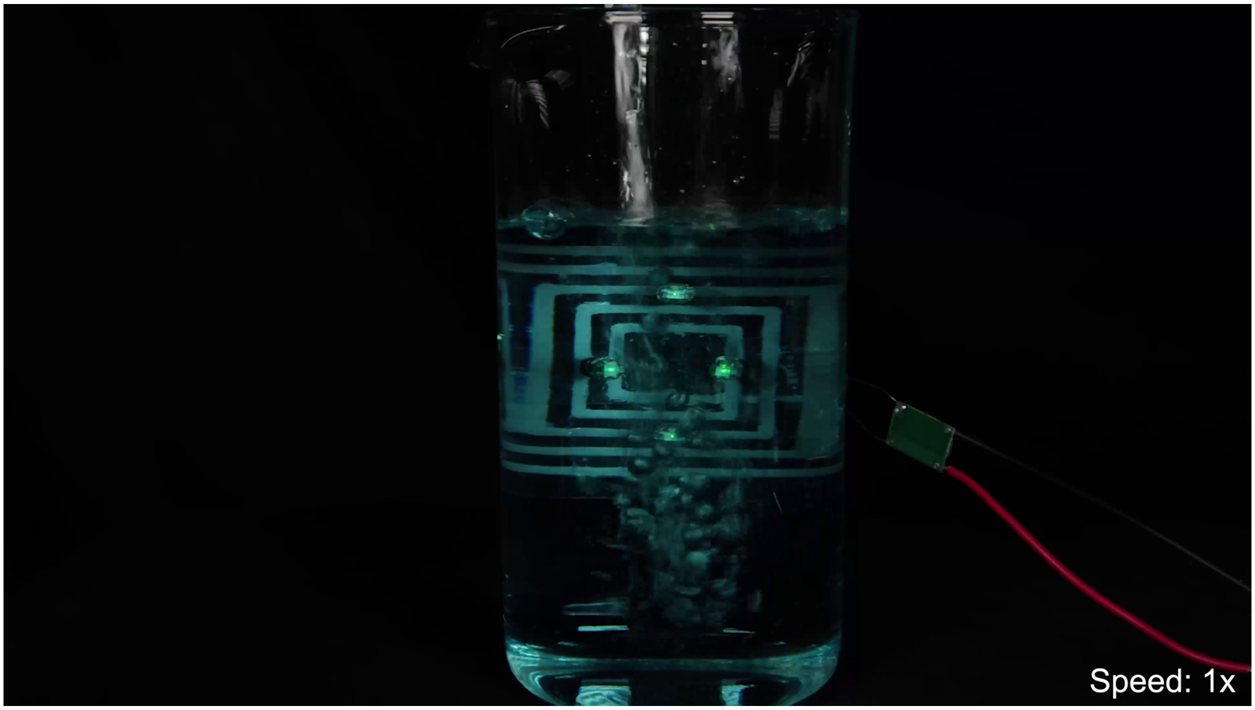

Movie S3 Powering LEDs wirelessly on a curved surface underwater.
